# Case of Recovery from Wound with Hernia of the Lung

**Published:** 1844-12-28

**Authors:** 


### CASE OF RECOVERY FROM WOUND WITH HERNIA OF THE LUNG.

BY DR. BARBIERI.

A boy, 13 years of age, fell from a tree upon the point of a hay fork, which tore up the right side of the anterior part of the chest between the fith and sixth ribs. The wound was transverse, about three inches in extent, and through this projected a considerable piece of the lung, also wounded in a transverse direction. There was a frequent cough with a gurgling sound in the bronchial tubes, but no bloody expectoration. The usual phenomena of a severe accident were present. The lung was reduced, the lips of the wound brought together by suture, and, by following an antiphlogistic regimen, the patient recovered without a bad symptom.—*Ibid, from L'Exp.*

